# Developmental Coordination Disorder and Most Prevalent Comorbidities: A Narrative Review

**DOI:** 10.3390/children9071095

**Published:** 2022-07-21

**Authors:** Federica Lino, Daniela Pia Rosaria Chieffo

**Affiliations:** 1 Clinical Psychology Unit, Fondazione Policlinico Universitario A. Gemelli IRRCS, 00168 Rome, Italy; federica.lino@policlinicogemelli.it; 2 Department of Life Sciences and Public Health, Catholic University of Sacred Heart, 00168 Rome, Italy

**Keywords:** developmental coordination disorder, comorbidity, ADHD, ASD, diagnostic profile

## Abstract

This narrative review describes, in detail, the relationships between Developmental Coordination Disorder (DCD) and most prevalent associated comorbidities in their complexity, heterogeneity and multifactoriality. The research has been conducted on the main scientific databases, excluding single case papers. Blurred borders between the different nosographic entities have been described and advances in this field have been highlighted. In this multifaceted framework a specific profiling for co-occurring DCD, ADHD and ASD signs and symptoms is proposed, confirming the need for a multidisciplinary approach to define new diagnostic paradigms in early childhood.

## 1. Introduction

Developmental Coordination Disorder (DCD) often mimics or co-occurs with other childhood disorders or conditions. Sometimes it could be difficult to set clear bonds from one condition to another. The boundaries between these disorders, especially in developmental age, can be blurred and more often conditions are correlated and collide together. As part of good clinical practice, researchers agree that clinicians should not focus solely on recognizing the main symptoms of a single disorder. It is always advisable to evaluate the associated problems in the best possible way, with a view to drafting ecologically valid rehabilitation pathways. A huge number of comorbidities has been described in literature for DCD such as Autism, Attention Deficit/Hyperactivity Disorder, Dyslexia, Dysorthography, non-verbal learning disorder, intellectual disability, Fragile x syndrome, Rett syndrome, Tourette syndrome, Tic disorder, Trisomy 21 and others. This paper focuses on most prevalent ones, pointing out the advances in this field of study.

## 2. Overview on Developmental Coordination Disorder

DCD is subcategorized as part of neurodevelopmental disorders and DSM V [1] defines the specific diagnostic criteria as follows:

(1) Motor coordination acquisition is below expectations for his or her chronologic age and clumsiness, inaccuracy of performance of motor skills or slowness are present;
(2) The motor deficit described in the previous criterion interferes with activities of daily living, academic achievements or leisure activities accordingly with the age;
(3) The onset of symptoms is determined at an early age;
(4) The motor deficit does not relate to a medical condition or disease.

The neural basis of DCD is still matter of research. The main deficits described are: voluntary gaze control during movement, training/dependent motor learning, internal modeling, cognitive/motor integration or atypicalities in motor network functioning. Recently a systematic combined review and meta-analysis explored behavioral and neuroimaging advances in research on DCD in recent years [2]; DCD prevalence in school-age population is about 6% [3]. The disorder refers to learning of movements and motor skills so present and important in everyday activities at developmental age; therefore, it is easy to understand how DCD also has consequences on the psychological side. Lack of self-esteem and reduced participation in play groups are described as usual in DCD population in addition to anxiety and mood deflection which can still persist to adult life [4,5,6,7,8].

It has been hypothesized that impaired mirror neuron function may have an influence on the generation of motor deficits in children with DCD. In particular, studies assume that a reduced activation of mirror neurons may contribute to a decreased capacity for internal representation and imitation of movements. Reynolds [9] has conducted some studies to investigate the possibility that a minimal underactivation of the mirror neuron system exists in adults and children who have DCD. A major difficulty in this type of study lies in the plastic capacity of the brain: Williams and colleagues [10] suggest that aging causes neuroplastic compensation mechanisms that modify networks dedicated to motor imagery.

Very often, DCD mimics or co-occurs with other childhood disorders or conditions and sometimes it could be difficult to set clear bonds from one condition to another.

## 3. Objective of the Current Review

Researchers have been exploring for years the features linking DCD with other clinical manifestations such as Autism Spectrum Disorders, ADHD and learning disorders. More recently, international research has given more space to a series of traits which occur in comorbidity with the diagnosis of DCD: greater attention was paid by researchers to ocular motility, neurovision deficits and the role of mirror neurons. The present review aims to examine most common comorbidities and traits in DCD.

## 4. Attention Deficit/Hyperactivity Disorder (ADHD)

Attention Deficit/Hyperactivity Disorder (ADHD) is one of the main developmental disorders whose prevalence is estimated to be around 4–9% of school-age children. More than 80% of the population diagnosed with ADHD has a comorbid condition [11]. ADHD is characterized by three CORE symptoms: inattention, impulsivity and hyperactivity. Along with this, difficulties in motor coordination are frequent. DCD is one of the most associated conditions with ADHD [12,13]. Some studies have been interested in evaluating the percentage of co-occurrence of the disorders, which, in a clinical sample, is estimated up to 50% [14,15], evaluating its effects on the level of functioning and on participation in motor activities in daily life [16,17]. Both the conditions of ADHD and DCD share a number of characteristics: children with ADHD often have difficulties in coordination and motor programming just as children with DCD show greater impulsivity and difficulties in inhibitory control. The scientific community has, therefore, questioned for a long time about possible commonalities from the etiological point of view, in the maturation and in the brain development of children with these two diagnoses. Recent studies have explored this issue and defined through neuroimaging that considerable differences are present both from the neurophysiological point of view and from a neurofunctional perspective, considering the neuronal circuits involved in the development of DCD and ADHD [18,19]. The motor difficulties identified in ADHD were investigated, highlighting a different trajectory of this deficit in the two studied populations [20]. Additional works focused on the longitudinal study of children with ADHD and DCD [21] emphasizing the importance of an early diagnosis. Researchers have also been interested in exploring the psychological well-being in children [22], adolescents and young adults with DCD and ADHD. Proxy report and self-report measures highlighted a worse perceived quality of life in these populations [23,24].

Coexistence of DCD and ADHD, has been described and termed in literature as “deficit of attention, motor control, and perception syndrome”. The definition suggests that there is a specific deficit profile on various levels in children with comorbidity [25,26]. As suggested by Irie and colleagues [27], research has shown that brain networks’ functioning in children who have DCD is different from that of children who have both DCD and ADHD. It is necessary for the progress of the research to highlight exclusion criteria in order to allow a deep knowledge of the cortical pathways through neuroimaging tools. Some studies made a step forward putting together DCD, ADHD and Autism Spectrum disorder populations to conduct some analysis on motor problems stability in early childhood [28,29]. Researchers suggest that screening of infants should include multiple domains and multiple time-points, since abilities development does not occur at a constant rate (reflecting different variables such as variation in growth, neuromuscular maturation, opportunity of practice, motivation and other factors) [30].

## 5. Autism Spectrum Disorder (ASD)

Within the range of developmental disorders, the Autism Spectrum Disorder (ASD) assumes a strong relevance as a comorbidity of DCD. Motor disorders are often pervasive in those diagnosed with autism, but comorbid diagnoses are unlikely. Some studies have investigated the link between motor coordination difficulties and ASD [31] and the debate among researchers is still open on the identification of DCD as a diagnosis that co-occurs with that of ASD or whether the motor deficits of autism should be considered typical of the disease [32,33,34]. In particular, patients with DCD share a poor motor competence and a difficulty in imitating movements with patients with ASD [35]. These difficulties, which are objectified in the DCD population in greater response times and greater number of errors, have been documented on both meaningful and non-meaningful gestures [36]. On the other side, the scientific literature has documented that, in contrast with ASD population, children with DCD can improve their motor skills through training [37,38,39,40].

Kilroy and colleagues [34] also investigated through the use functional magnetic resonance the AON (Action Observation Network), a brain area thought to be involved in the imitation of movements, coming to the conclusion that in autism there is not a direct correlation with neurobiological dysfunction. Some other studies recently investigated the social skills of children with DCD, describing them on a continuum between children with normotypic development and children with ASD [41]. In a similar scenario, a dual-track research line would be appropriate, to explore on the one hand different etiopathological phenotyping clusters in DCD (to better understand the deficits in motor imitation) and on the other, an implementation of clinical pathways focusing on motor difficulties to strengthen intervention programs where necessary.

## 6. Language Disorders

Several studies have been interested in studying the relationship between DCD and specific language disorders. Some authors have pointed out that a significant percentage of children with specific language disorders would indeed qualify for a diagnosis of DCD [42]. This should be of crucial importance for clinicians who are invited to implement broad spectrum assessment protocols for young children with specific language disorders. In particular, some studies have focused on determining whether speech-language impaired toddlers are more at risk of developing DCD as well. The results indicate that once they arrive at the kindergarten, a good number of these children have motor difficulties consistent with DCD diagnosis [43]. A delay in motor difficulties onset is probably due to the developmental challenges that involve gradually fine-motor control and the daily new challenges imposed by growth. The fact that speech-language impaired toddlers are at increased risk of developing DCD opens up a number of questions from a diagnostic and rehabilitative point of view. Studies that explore DCD in populations below school age are very rare. It would be very valuable to obtain more data on this age group to replicate the aforementioned results and define comprehensive diagnostic and rehabilitation paradigms in early childhood.

## 7. Dyslexia and Dysgraphia

Among the best-known comorbidities of DCD, there is Dyslexia, a reading disability consisting of a significant difficulty with speed and accuracy of word decoding. Dewey and colleagues show a difference in evidence of attention, learning and social adaptation in subjects with DCD compared to control subjects [44]. A series of research confirms that children with DCD have a deficit in predictive motor control [45,46]. Motor coordination, eye movements’ control and implicit motor learning difficulties are however often discussed in the literature even in the case of Dyslexia. Cignetti and colleagues identify a common deficit of the feedforward component of motor control in children with DCD and in children with dyslexia compared to control groups in a bimanual unloading task [47]. Bellocchi and colleagues recently explored the visual-attentive process in a reading task for children with dyslexia and DCD and studied the impact of the co-occurrence of both conditions on reading processes. In conclusion, the authors found no results to support the cumulative hypothesis: comorbidity does not add to the severity of the cognitive deficit. Study results demonstrate that visual attentional processing in word recognition is not impaired in children with isolated DCD or comorbid DCD. This latter point seems to have particular relevance in the clinical field when it comes to the need to define specific profiles for children with isolated dyslexia or comorbid DCD with a view to drafting a personalized treatment [48]. On the other hand, Maziero and colleagues highlight how, in a working memory paradigm, children with dyslexia perform worse in verbal memory tests and children with DCD perform worse in a spatial working memory test. Children with dual diagnoses perform worse in both tasks [49].

Beyond the motor deficits, about half of all children with DCD show difficulty in learning to write [50,51]. Dysgraphia is a disturbance in the production of written language, specifically related to the mechanics of writing. Difficulties can arise in the very early years of school when the child tries to put letters together and can greatly impact the child’s ability to write correctly. Lopez and colleagues identified the association between dysgraphia and minor neurological dysfunction in a sample of DCD subjects, revealing that in this population the presence of dysgraphia could be linked to specific conditions [52].

## 8. Ocular Motility

In order to acquire proper motor skills, adequate visual feedback is necessary. Rafique and colleagues recently investigated the effects of accommodation anomalies which contribute to motor skills impairment [53]. Some atypicalities in ocular motility have been identified in patients with DCD [54], in particular with regard to sustained engagement on fixation (deficient in subjects with DCD) and differences in gaze behavior compared to control groups [55,56]. Gaze training was investigated in order to verify whether it caused, retrospectively, benefits in the organization of the movement [57]. The first results in this field of study seem to confirm the effectiveness of the training. Some researchers have also been interested in the effects of a gaze training combined with psychosocial training [58]. The outcomes of such a paradigm were evaluated through results in motor exercises and proxy questionnaires: the study provided positive results from a motor point of view but not only. In fact, gains were described also in terms of aptitude and motivation to exercise sports. Miles and colleagues explored more specifically which type of training children with DCD could benefit from, suggesting in Quiet Eye Training (QET) an effective integration to traditional training [59]. Today the number of studies examining ocular motility in subjects with DCD is still small, but there is an initial number of studies supporting the fact that it is a domain worth exploring in more depth.

## 9. Emotional Disorders

In recent decades, the number of studies investigating emotional consequences of DCD has increased [60,61,62,63,64,65]. What research has shown so far is an increase in anxiety, a decline in mood and a lowering of perceived self-efficacy levels [66]. From a family perspective, a perception of a lack of support is emerged, which is a major stressor [67]. Some other studies have emphasized the link between mental well-being and some psychosocial factors such as bullying, the ability to build friendships and good self-esteem. This type of result reinforces the idea that prevention in the psychological and social field could be a key factor in the treatment of DCD and its comorbidities. Families who are diagnosed with a DCD child, experience significant difficulties. In a recent study, Licari and colleagues proved that many families receive a diagnosis only after years [68]. In addition, since a very high percentage of families undertake a therapeutic pathway for motor difficulties, families often experience a case-by-case variable economic stress.

## 10. The Importance of an Early Intervention

In the present study, we investigated the most frequent comorbidities associated with DCD. As mentioned in the introduction to this paper, studies on the comorbidities of DCD have explored other clinical conditions as well. As DCD has been investigated over the years, researchers’ interest has widened also to the study of the co-occurrence of other conditions, such as genetic syndromes [69,70,71,72,73] and Tic disorders [74,75,76,77] (with which the DCD shares membership in the motor disorders subcategory of DSM 5). The reason why researchers have widened their interest to these conditions, which will not be covered here, is that typical DCD traits are present in a long series of clinical conditions. It is desirable that future research will deepen our understanding in these areas of knowledge to better understand underlying etiological factors in early childhood.

In recent years, the need for early diagnosis has emerged with increasing force [78]. Some studies have focused on interventions dedicated to children at risk of developing a diagnosis of DCD in small groups and others on entire class groups [79,80,81,82]. Some of these interventions have considered not only a child training but also an educational component for parents and teachers which is defined as a crucial point to allow the child to overcome difficulties [83,84,85]. Since pre-term born children are considered to be at increased risk of developing DCD, some studies have focused on this population [86,87]. The number of studies that have considered treatments dedicated to children at risk of developing DCD or diagnosed with DCD under the age of 5 is, nevertheless, particularly small. Some of these studies suggest the engagement of these children in activities that require repetitive movements, such as swimming or martial arts. In addition to participation, these children must be provided with support in learning the right movements [88].

## 11. Discussion

DCD has a significant percentage of comorbidities, in particular neurodevelopmental disorders. The scientific community is currently investigating the links that bind the various clinical conditions by identifying neuroanatomical and neuro-functional connection circuits, highlighting the commonalities and differences. In developmental age, more than in adulthood, the boundaries between the various disorders are often blurred.

In some cases, as in that of language disorders in toddlers, it is possible to detect a greater longitudinal risk of development of DCD typical disorders. This seems to be a crucial age in DCD. The very early signs and symptoms detected in this time window could be intercepted and assigned to therapeutic pathways. In spite of the increasing number of studies on DCD in school-age, only a small number of studies investigated the disorder in early childhood. More research is needed in this age group to create more sensitive and comprehensive diagnostic and rehabilitative models exploring symptoms at the very early onset. Emerging evidence shows that children, in particular those at greatest risk of DCD, may be identified before formal school entry. Earlier diagnosis will allow for earlier intervention, which may help to improve the developmental trajectories of children. Moreover, secondary symptoms of the DCD would be reduced. In the words of Zwicker and colleagues it is recommended that health care providers explicitly use the term ‘at risk of DCD’ [78]. This would help in undertaking screenings and early diagnosis.

Based on the results emerged in literature and in the light of the strong comorbidity between motor and attentional deficits typical in DCD, ASD and ADHD, it seems advisable to define specific profiles in clinical settings taking care of commonalities and allowing a specific classification for this condition. The concept of “Deficit of attention, motor control, and perception syndrome” could be amplified and not only related to DCD and ADHD. It is necessary to create specific profiles making possible to trace a more precise functioning of the child inside and outside of the clinical setting.

What emerges from the analysis of the various comorbidities is the need to identify multidimensional and multidisciplinary diagnostic protocols and tools to highlight the weak points and strengths of individual patients’ profiles in early childhood. As Gillberg asserts in his article on early symptomatic syndromes, the presence of relevant difficulties in a domain such as language, general development, motor coordination, attention, sociability, activity, behavior, mood and sleep for children aged between 3 and 5 is a precursor of difficulties in the same domain or precursor of overlapping difficulties in another domain years later [89].

This must be taken into account if we want to define effective therapeutic opportunities.

The need to implement treatment paradigms that also consider a psychoeducational perspective has also emerged. This is intended, on the one hand, to sensitize psycho-social professionals to DCD characteristics and on the other hand to make children and families feel understood and supported. Recently, some researchers conducted a systematic scoping review which gave birth to guidelines focusing on family-centered care, communication with, and providing information to parents with young children with developmental disabilities considering ADHD, DCD and ASD [90].

## 12. Conclusions

This contribution describes, in detail, the relationships between DCD and associated comorbidities in their complexity, heterogeneity and multifactoriality. In such a scenario, the need for a multidisciplinary approach both for the diagnosis and for the clinical management is confirmed.
